# Procedure Time for Gastric Endoscopic Submucosal Dissection according to Location, considering Both Mucosal Circumferential Incision and Submucosal Dissection

**DOI:** 10.1155/2016/9183793

**Published:** 2016-12-19

**Authors:** Hironori Konuma, Kenshi Matsumoto, Hiroya Ueyama, Hiroyuki Komori, Yoichi Akazawa, Misuzu Ueyama, Yuta Nakagawa, Takashi Morimoto, Tsutomu Takeda, Kohei Matsumoto, Daisuke Asaoka, Mariko Hojo, Akihito Nagahara, Takashi Yao, Akihisa Miyazaki, Sumio Watanabe

**Affiliations:** ^1^Department of Gastroenterology, Juntendo Nerima Hospital, Tokyo, Japan; ^2^Department of Gastroenterology, Juntendo University School of Medicine, Tokyo, Japan; ^3^Department of Gastroenterology, Juntendo Sizuoka Hospital, Sizuoka, Japan; ^4^Department of Human Pathology, Juntendo University School of Medicine, Tokyo, Japan

## Abstract

*Background*. Previous assessments of technical difficulty and procedure time for endoscopic submucosal dissection (ESD) of gastric neoplasms did not take into account several critical determinants of these parameters. However, two key phases of ESD determine the total procedure time: the mucosal circumference incision speed (CIS) and submucosal dissection speed (SDS).* Methods*. We included 302 cases of* en bloc* and R0 resection of gastric neoplasms performed by 10 operators who had completed the training program at our hospital. Twelve locations were classified based on multiple criteria, such as condition of surrounding mucosa, lesion vascularity, presence of submucosal fat, ulcers, scars, fibrosis, and scope and device maneuverability. Lesions in different locations were classified into three groups based on the length of the procedure: fast, moderate, or late.* Results*. A significant difference was found in CIS and SDS for each location (*p* < 0.01), which demonstrates the validity of this classification system. In several locations, CIS and SDS were not consistent with each other.* Conclusion*. CIS and SDS did not correspond to each other even for lesions in the same location. Consideration of ESD procedure time for gastric neoplasms requires a more elaborate classification system than that previously reported.

## 1. Introduction

The endoscopic submucosal dissection (ESD) technique was introduced to facilitate* en bloc* resection of early gastrointestinal neoplasms, which allows for precise histological diagnosis and minimizes the chances of recurrence [1, 2]. The popularity of ESD has rapidly increased in Asia and the rest of the world. Guidelines for ESD have been developed recently in Europe and the USA [3, 4].

However, the procedural complexity of ESD, especially for gastric neoplasms, tends to vary with the lesion location and vascularity, presence of ulcers, scars, and fibrosis. These factors also determine the risk of intraoperative complications such as perforation and catastrophic hemorrhage, more so in inexperienced hands. Therefore, the success of ESD, to a large extent, depends upon an in-depth understanding of the specific attributes of the lesion.

Recently, several studies have examined the technical challenges in performing ESD with respect to lesion location and procedural time. Scarred and undifferentiated lesions as well as those located in the upper third of the stomach were reported to be typically challenging and required more time [5, 6]. However, the classification of lesions based on location alone (namely, upper, middle, and lower) does not take into account the other determinants of procedural complexity while performing ESD [5, 7, 8]. For example, during circumferential incision at the greater curvature, the mucosa is thick and therefore harder to cut, and the bleeding is more than at other sites. Furthermore, ulcers, scars, and submucosal fibrosis (referred to as “hidden fibrosis” in this paper) tend to occur more commonly along the lesser curvature. Further, submucosal fibrosis is often detected only intraoperatively. Thus, in our opinion, the indicators for actual treatment difficulty or procedure time have been neglected in the classification methodology used in previous reports. Moreover, there are two critical phases that define the procedural complexity (and hence procedure time) for ESD: (1) the mucosal circumference incision phase (CIS) and (2) the submucosal dissection phase (SDS). We believe that these two aspects merit separate consideration.

In this study, all the situations in which procedure time was considered to be different are discussed and classified as new locations. Furthermore, the procedure time for each location is examined with regard to both the mucosal CIS and SDS.

## 2. Materials and Methods

### 2.1. Selection Criteria for ESD Operators

**Figure 1:**
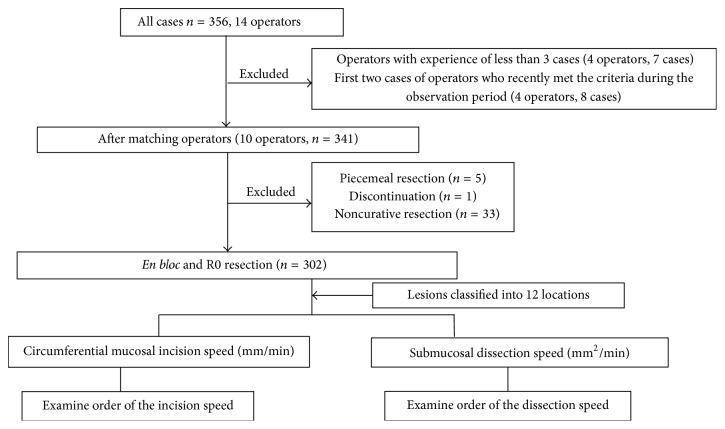
Study outline. Operators newly enrolled during the observation period and their first two cases.

According to the training standards proposed by Tsuji et al. [9], ESD operators must have an experience of a minimum of 1,000 cases of upper gastrointestinal endoscopy, with 40 cases or more involving ESD assistance and 20 cases or more that required post-ESD prophylactic hemostasis at the ulcer site. Motivated by this training system to introduce ESD with greater safety, endoscopists at our institution observe and assist in ESD procedures for 1 year (approximately 150 cases). Subsequently, they train on pig models for a minimum of 10 procedures. Endoscopists who have reached the level at which they no longer accidentally puncture are allowed to practice on humans. Furthermore, to minimize any difference in skills between operators, the inclusion criteria for enrollment in the present study consisted of operators who had performed the procedure on at least three humans with variations in procedure time in up to two procedures. For operators that were newly enrolled during the observation period, only those who met these criteria were included (Figure 1).

### 2.2. Target Lesions

Differentiated type, undifferentiated type, and mixed type (differentiated and undifferentiated) with an undifferentiated component of <20 mm were used according to the expanded criteria of ESD [7, 10, 11].

Adenomas that were considered precancerous included the following: (1) lesions >20 mm in diameter, (2) those with a depression, (3) those with rapid growth in a short time, and (4) those showing high-grade atypia on biopsy [12–14]. Furthermore, neuroendocrine cell tumors considered endoscopically curable and large benign polyps that are difficult to treat via endoscopic mucosal resection (EMR) due to risk of bleeding were also included in the present study, if treated with ESD.

### 2.3. Clinical Study of ESD

All cases of undifferentiated mixed type gastric cancer between April 1, 2009, and July 31, 2014, in which ESD was indicated as per the Japanese guidelines were reviewed [7, 11]. A total of 341 gastric ESD patients with 356 lesions were identified. Among these, operators and their patients who did not meet the selection criteria were excluded. Further, the first two cases of operators who recently met the criteria during the observation period were also excluded. Finally, piecemeal resection, discontinuation, and noncurative resection cases were also excluded to include only curative* en bloc* and R0 resection cases in the study (Figure 1).

### 2.4. Treatment Area Classification, Resected Lesion Circumference, and Area Calculation Method

**Figure 2:**
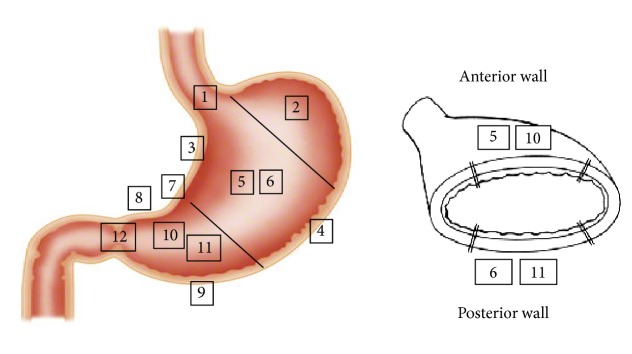
Twelve locations divided according to the consideration of a variable situation. 1: the lesion across the esophagogastric junction (AEGJ); 2: fornix; 3: lesser curvature of the body; 4: greater curvature of the body; 5: anterior wall of the body; 6: posterior wall of the body; 7: lesion across the angle; 8: lesser curvature of the antrum; 9: greater curvature of the antrum; 10: anterior wall of the antrum; 11: posterior wall of the antrum; 12: lesion across a pylorus ring (APR).

Prior to the observation period, six operators who met the criteria discussed each of the listed items for which the procedural complexity was expected to differ. These included the mucosa (pyloric, fundic, and cardiac areas), state of the submucosa (vascularity, ulcers, scars, and hidden fibrosis observed for the first time at dissection), and maneuverability of the endoscope and device when different from the type of scope used normally. On the basis of this discussion, a total of 12 locations were identified (Figure 2). Representative cases of these variations are shown in Table 3.

The 12 locations were as follows: 1: lesion at the esophagogastric junction (AEGJ); 2: fornix; 3: lesser curvature of the body; 4: greater curvature of the body; 5: anterior wall of the body; 6: posterior wall of the body; 7: lesion across the angle; 8: lesser curvature of the antrum; 9: greater curvature of the antrum; 10: anterior wall of the antrum; 11: posterior wall of the antrum; and 12: lesion across the pylorus ring (APR) (Figure 2). And we examined the proof of the validity of this taxonomy statistically.

Resected specimens were obtained by placing markings for the incision line 10 mm outside the lesion; then resection was performed outside this line for undifferentiated and mixed type lesions. For all other cases, markings for the incision line were placed 5 mm outside the lesion margin, and the incision was made outside this line. As the resected specimens were oval, the area and circumference were calculated from the long and short axes. Furthermore, the CIS per unit length (mm/min) and SDS per unit area (mm^2^/min) were calculated for each location. Lastly, these were divided into three groups according to the median resected length/min and the median unit area for each location from the CIS and SDS in order of size. Next, these groups were further stratified according to procedure time (fast, moderate, and late groups).

### 2.5. ESD Procedure

The main endoscope used was GIF Q260J (Olympus, Tokyo, Japan); in duodenum bulb, a reverse maneuver is not possible with the GIF-Q260J, so, in all lesions of duodenum bulb, we used GIF-Q260 (Olympus, Tokyo, Japan). When a closed approach was difficult, the endoscope was changed to GIF 2TQ260M (Olympus, Tokyo, Japan). For the injection solution, a mixture of normal saline with 1% indigo carmine dye was used. In the event of poor uptake, an adequate amount of sodium hyaluronate with high viscosity was used. For basic techniques, we performed a precut in the region of the mucosa using a dual knife (KD-650, Olympus, Tokyo, Japan). Then, a mucosal circumferential incision was made using the dual knife or insulation-tipped (IT) knife 2 (KD-611L, Olympus, Tokyo, Japan). Submucosal dissection was performed using the IT knife 2 and/or a dual knife (especially if a dual knife was used for the scar tissue). In the event of active bleeding or if prominent blood vessels were present, hemostasis was ensured using a coagrasper (FD-410LR, Olympus, Tokyo, Japan). A high-frequency surgical unit for cutting and coagulation (Erbotom VIO300D, ERBE, Tubingen, Germany) was employed.

### 2.6. Definition

Curative resection was defined as per the expanded criteria of ESD [7] in the case of an R0 and* en bloc* resection. Ulcers and scars that were observed on preprocedural endoscopy were represented as an “ulcer or scar.” Fibrosis first observed in the submucosal layer at the time of treatment was recorded as “hidden fibrosis.” Instances where hemostasis was required more frequently than usual or when preincisional coagulation was required due to presence of several submucosal blood vessels were defined as “much time to hemostasis.” Tumor morphology was expressed according to the Paris classification [15], and pathological findings were documented as per the Vienna classification [16].

This study was conducted in accordance with the Declaration of Helsinki and was approved by the Institutional Review Boards at Juntendo University Hospital.

### 2.7. Statistical Analysis

Interquartile range (IQR) was calculated to determine variations in incisional circumference length (per unit length) for each location and the dissected area. With regard to operator skill differences for each location, large deviations were marked with an “*x*.”

Outliers were defined as “cases exceeding 1.5 times the interquartile range above the third quartile.” Data pertaining to categorical variables are presented as constituent ratios. Between-group differences in case of normally distributed variables were assessed using one-way Analysis of Variance; nonnormally distributed variables were assessed using Kruskal-Wallis or Steel-Dwass tests, as appropriate. Fisher's exact test or *χ* ^2^ test was used for all the other analyses. Odds ratios, absolute differences, 95% Confidence Intervals (CI), and* p *values are reported. Statistical significance was defined as *p* < 0.05. All statistical analyses were performed using SASS version 9.4 (SAS Institute, Cary, NC, USA).

## 3. Results

### 3.1. Results of the Validity of 12 Locations Classification

In this classification system, we found a significant difference between different groups for all CIS and SDS in each location, demonstrating the validity of this classification method (*p* < 0.01) (Table 4).

### 3.2. Study Outline

A total of 10 operators participated in the study: six operators who completed the training program prior to the observation period and had experience performing ESD on at least three patients, plus four newly added operators with ESD experience of at least three patients. On the basis of operator adaptations, 341 lesions remained for 10 operators. Furthermore, we excluded five cases that finally became piecemeal and snaring resection. In addition, we excluded one case in which the procedure was discontinued. Among the cases in which* en bloc* resection was performed, 33 noncurative resections (lesions invading the submucosa and positive lymphovascular invasion, positive margins, or expanded indication [7]) were excluded. Therefore, a total of 302 lesions treated by curative resection (*en bloc* and R0 resection) were included in the analysis (Figure 1).

### 3.3. Baseline Clinical Results

A total of 302 lesions were examined in the study; the male-to-female ratio was 2.5 : 1. Macroscopically, flat, and depressed types accounted for >95% of the total lesions. The median tumor diameter was 11 mm, and the median size of the resected specimen was 34 mm. The most common histological tumor type was differentiated adenocarcinoma (81.8%); mixed types containing a differentiated type and ≤20 mm undifferentiated types accounted for 4.0%; lesions of an undifferentiated type accounted only for 1.7% of the total number of lesions. Adenomas represented 11.9% of the cases. In addition, there was one case of a neuroendocrine tumor and one of a hyperplastic polyp. Ulcers or scars were confirmed in 7.9% of cases, and hidden fibrosis in 11.6%. Moreover, much time to hemostasis was observed in 16.9% of the cases. Complications involving perforation occurred in 2.3% and delayed bleeding in 3% of cases, comparable to results that have been reported elsewhere (Table 1) [1, 2, 17–19].

### 3.4. Results for Each Classified Location

The breakdown of the number of cases according to 12 classified locations is shown in Table 2. The lesions were most commonly found on the posterior wall of the gastric body (*n* = 46 [15.2%]), while those extending to the pyloric ring were least common (*n* = 4 [1.3%]). The clinicopathological characteristics by lesion location are listed in Table 3. No significant between-group difference was observed with respect to age (*p* = 0.24). Ulcers or scars exceeded 10% in four locations including the following: (1) AEGJ, location 1 (20%); (2) fornix, location 2 (14.3%); (3) angle, location 7 (11.8%); and (4) greater curvature of the antrum, location 9 (11.8%).

**Figure 3:**
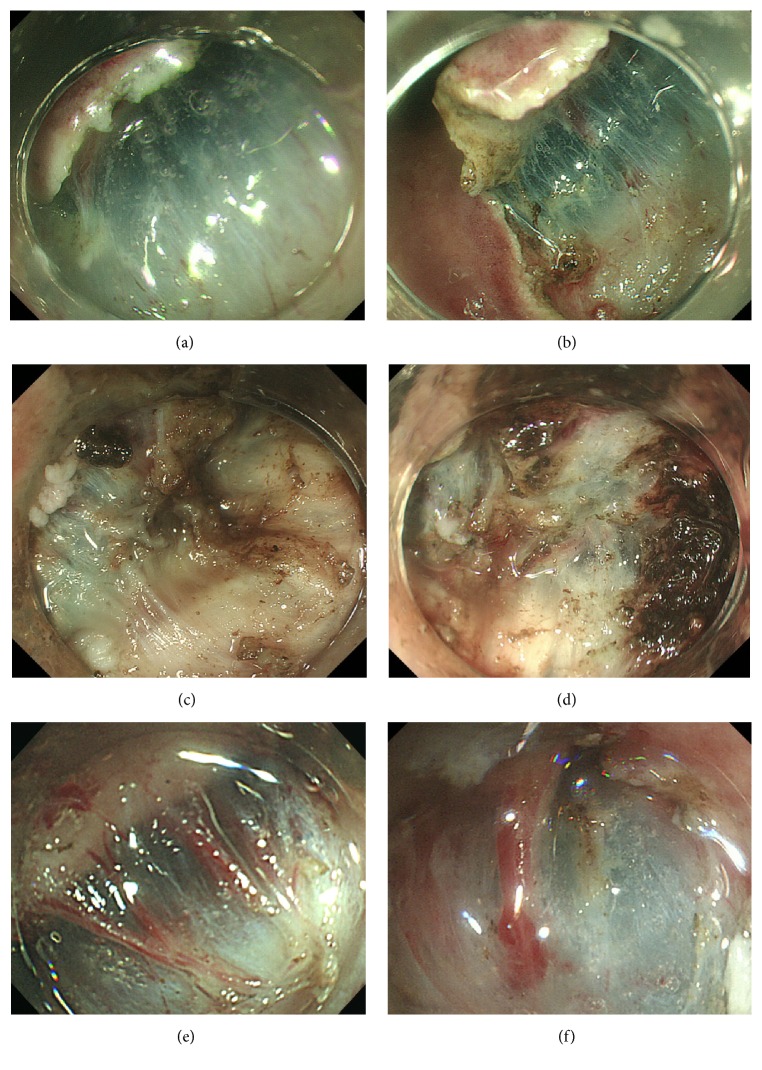
Variations of lesser curvature. (a, b) Easy case of submucosa. (c, d) “Hidden fibrosis.” (e, f) Many perforating vessel case.

A high rate of hidden fibrosis was observed in fornix, location 2 (28.6%), and the lesser curvature of the body, location 3 (24.2%) (Figure 3). In addition, other locations in which fibrosis exceeded 10% were the posterior wall of the body, location 6 (17.4%), and the lesions across the angle, location 7 (17.6%). It is important to note that a high rate of fibrosis of 24% was observed in the lesser curvature of the body (location 6), despite the fact that ulcers or scars were found in only 6.1% of these cases.

Much time to hemostasis was in the following, in descending order: (1) AEGJ (location 1), 60%; (2) fornix (location 2), 42.9%; (3) lesser curvature of the body (location 3), in 30.3%; (4) posterior wall of the body (location 6), 28.3%; (5) lesion across the angle (location 7), 26.5%; (6) anterior wall of the body (location 5), 22.7%; and (7) greater curvature of the body (location 4), in 14.3%, with a higher rate at the lesser curvature of the body (location 3) than at the greater curvature of the body (location 4).

Table 3 shows that a significant difference was observed in the tumor diameter between each location (*p* < 0.05). Furthermore, a significant difference was also observed for the circumference of the resected specimen or the area of the resected specimen between each location. Overall, the variations in the values were statistically significant (*p* < 0.01).

**Figure 4:**
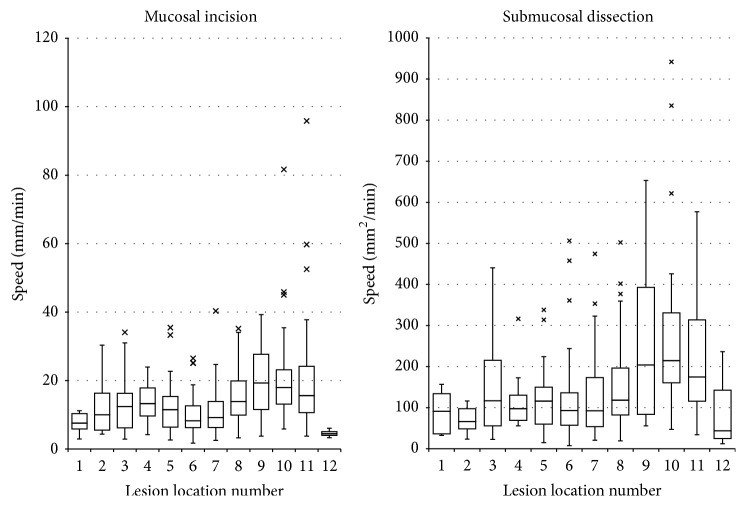
Interquartile range of the mucosal incision and submucosal dissection speed. This figure shows a graph of the mucosal incision and submucosal dissection speed of each of the 12 locations. By noting the interquartile range, the variation in the rate was clear.

Table 4 shows the variations in the resection time in descending order of size of the median circumference incisional length per minute and median dissected area of the submucosal layer. The IQR for each location is graphically presented in Figure 4. A significant variation in overall CIS and SDS was found by location (*p* < 0.01).

Regarding CIS, while the speed was faster for lesions of the antrum than for those at other locations, it was slowest for the antral area along the lesser curvature, location 8 (median: 13.9 mm/min). The overall incisional speed was slower for the gastric body than for the antral area and was the slowest for location 6, the posterior wall of the gastric body (median: 8.2 mm/min). The second slowest location (i.e., 11th position) was location 1, AEGJ (median: 7.6 mm/min), and the slowest location was location 12, APR (median: 4.5 mm/min).

In contrast, the greatest IQR was found for the antral area along the greater curvature, location 9. Although the incisional speed was slow in the AEGJ and APR, the IQR was small, with little variation in speed.

With respect to SDS, as expected, the overall speed was fast for antral lesions; however, when dissecting areas around the antrum, the speed was slowest for location 8, the lesser curvature of the antrum (median: 118.2 mm^2^/min). The antrum exhibited a large IQR, and, overall, the speed tended to vary greatly at the same sites of the antrum. The variation was particularly high in location 9, the greater curvature of the antrum. For the gastric body, the speed was the slowest for location 6, the posterior wall, similar to the case for CIS. However, the greatest variation in the dissection speed was observed for location 3, the lesser curvature (IQR: 159.40). The SDS for the AEGJ, APR, and fornix was lower than that for other locations.

The second slowest SDS (i.e., the 11th position) was the fornix, and the slowest was for the APR. The fornix was at the eighth place with respect to CIS but at the eleventh place for SDS. The greatest IQR for SDS was observed for location 9, the greater curvature of antrum (IQR = 309.3).

### 3.5. Grouping of CIS and SDS for Each Location

The results are shown in Table 5. The fornix had the fifth highest speed for CIS, placing it in the moderate group, whereas, for SDS, it was the second slowest overall, placing it in the late group. Furthermore, the time required for CIS at the posterior wall of the gastric body was the third overall, placing it in the late group. However, for SDS, the dissection speed was fifth overall, placing it in the moderate group, similar to other lesions of the body. For both APR and AEGJ, CIS and SDS were slow, placing them in the late group.

With regard to CIS, a significant difference was observed between the fast and moderate groups and between the moderate and late groups (*p* < 0.01). For SDS, no significant difference was observed between the moderate and late groups (*p* = 0.42). However, a remarkably high difference was observed between the fast and moderate groups (*p* < 0.001) (Table 5).

## 4. Discussion

With regard to the ESD difficulty by location, the longest reported procedure time has been reported for the upper third of the posterior wall [6, 19]. However, these previous studies did not take into account other factors that tend to vary with the location of the lesions; these include technical considerations (e.g., device angle and scope maneuverability) and lesion characteristics (e.g., vascularity, ulceration, scarring, fibrosis, characteristics of contiguous mucosa, and submucosa). Furthermore, the determinants of CIS and SDS are distinct, but procedural complexity in previous studies was only assessed with respect to overall procedure time.

In the present study, unlike conventional location classification methods, we incorporated several key variables that determine procedural complexity. Additionally, we also examined CI and SD as separate factors. Using this classification system, we found a significant difference between different groups for all CIS and SDS in each location, demonstrating the validity of this classification method (*p* < 0.01) (Table 4).

Undifferentiated lesions have been reported to be more difficult to assess [6]. This may be explained by the fact that the differentiated type is covered by a thin layer of atrophic mucosa, which makes it relatively easy to dissect the mucosa and submucosa. In contrast, the mucosa surrounding the undifferentiated type tumor is rarely atrophic. This may be attributed to the fact that the incisional diameter may have been much larger than the actual lesion because of rich mucosal vascularity. Moreover, in lesions with the presence of a large number of blood vessels and fat in submucosa, the incisional line was marked approximately 10 mm away from the lesion. As shown Table 3, at every location, there were significant differences in tumor size (*p* < 0.5). But actual resected specimen sizes are variable. For that reason, when the tumors were of an undifferentiated or mixed type, there was a need to make a larger excision. Endoscopically, when there is an ulcer or scar in order to allow the scope access to the under mucosa a larger than normal incision must be made, this may have resulted in the more significant difference in circumference (mm) and resected area (mm^2^) (*p* < 0.01). If the procedure were to take place in area with a large amount of blood vessels with factors written above taking effect, the time needed to stop the hemorrhage and much time to hemostasis would prolong the procedure time. In the case of an ulcer or scar, the time needed to excise the scar in addition to the larger than normal incision would also result in a longer procedure time. Hidden fibrosis can only be known at the time of dissection; thus it can said that it has no correlation with the factors mentioned above.

Regarding the overall ESD, the incisional and dissection speed was faster in the case of antral lesions than those for other locations. However, the IQR tended to vary greatly during the actual dissection (Figure 4). We believe that this was due to the relative ease of dissection in this region; there were large differences in the performance of experienced and inexperienced operators despite our stringent operator selection criteria. However, for the procedures involving the antrum, both the incision and dissection speeds at the lesser curvature for CID and SDS were slower than those of other antral sites. We believe that this was because, in the lesser curvature of the antrum, the endoscopic device and lesion can readily become perpendicular to each other, and the lesion site may be relatively difficult to approach with the scope in this position. In locations other than the antrum, particularly the AEGJ and fornix, ESD tends to be challenging even for experienced operators, which may be responsible for the relatively low variability in the procedure time.

The SDS varied greatly in lesions in the lesser curvature of the body, relative to that in other locations of the body (IQR = 159.4). The gastric angle and lesser curvature of the body are sites in which ulcers and scars are common. However, in the lesser curvature of the body, hidden fibrosis is often incidentally discovered during endoscopic dissection, regardless of the lack of scars; of note, we also found fibrosis in 24.2% of the dissections in this area in the present study. Therefore, increased time is required for detachment when the submucosa is not sufficiently lifted by local injection. Furthermore, the lesser curvature of the body can be difficult to approach with a scope or device depending on the shape of the stomach. Moreover, some lesions were viewed perpendicularly, which may have been responsible for the high variability in the incisional speed among lesions located at the same gastric body site. Moreover, it was initially believed that the greater curvature had more blood vessels and hemostasis. However, in clinical practice, more cases of much time to hemostasis were observed for the lesser curvature lesions (30.3% versus 14.3% for the greater curvature lesions) (Table 3).

While increased perilesional vascularity in the case of ulcers, scars, and hidden fibrosis is believed to be another potential reason for the occurrence of many blood vessels, several perforating branches of blood vessels to submucosa in the lesser curvature are also contributory factors. However, a difference in the presence or absence of blood vessels and fibrosis in the lesser curvature greatly affects the procedure; therefore, it is not straightforward to predict the time required for dissection. Consequently, we suggest that expert operators with experience in difficult situations always be prepared to take turns at any time.

Differences in CIS and SDS between the fast, moderate, and late groups were found in the fornix (location 2) and in the posterior wall of the body (location 6). The fornix was classified in the moderate group for CIS, but the late group for SDS. This is because the scope and device end up perpendicular to the lesion and thus it is difficult to maneuver horizontally as both the submucosa and muscularis were thin.

The posterior wall of the body was classified into the moderate group for SDS, but the late group for CIS. We believe that this was attributed to the fact that reverse maneuver was considered to be difficult for the circumferential incision, in addition to the fact that there were more large blood vessels and fat in the mucosa and submucosa than in the other sites. However, upon completion of the circumferential incision, not as many blood vessels or as much fat was observed in the submucosa as that at the time when the circumferential incision was performed, and, therefore, dissection was considered easier than the circumferential incision. Locations that became classified into the late group for both CIS and SDS included the following: location 1, AEGJ; location 7, across the angle; and location 12, APR.

Maneuverability for the AEGJ (location 1) is considered to be poor due to the following reasons: hemostatic treatment is difficult due to several palisade blood vessels traversing the submucosa; dissection is difficult due to inflammatory adhesions caused by gastroesophageal reflux disease or other conditions; the working space on the oral side of the lesion is narrow due to the requirement for the intraesophageal maneuver; and reverse maneuver on the anal side is difficult to move the scope closer to the lesion. The angle (location 7) has a sharp anatomical bend and, therefore, must be approached from various angles. It is also a common site for ulcers and scars, which cause adhesions and render the dissection more challenging. APR requires resection of the pylorus ring (location 12) and reverse maneuver in the narrow bulbous working space as well as precautions for prevention of duodenal perforation. Consequently, it was assumed to be the location in which numerous techniques were most required. For the reasons provided above, in APR, reverse maneuver was not possible with the GIF-Q260J typically used. In all cases, the scope had to be changed to GIF-Q260, which has the greatest flexibility in the tip structure. Furthermore, to ensure the working space for this location, it is important to use a scope without the tip hood mounted so as not to impede inversion.

Initially, we believed that the greater curvature belonged to the late group for both the CIS and SDS, on the basis of the mucosal thickness, blood vessels, and the amount of fat. However, in our analysis, it was found to belong to the moderate group.

Complications involving perforation occurred in 2.3% and delayed bleeding in 3% of cases, comparable to results that have been reported elsewhere (Table 1) [1, 2, 17–19]. However, our hospital is a specialized center for ESD; many cases including lesions with high difficulty and high complications have been treated at our hospital. Thus, we believe that such a condition is rather rare compared with that at other hospitals.

The limitations of this study include the fact that it was a single-center study, and there may be a bias according to the endoscopist who performed the ESD.

In the present study, a more detailed classification of the resection sites was used than those that have been previously described; this inevitably reduced the sample size for each lesion location. However, a larger sample size would have made it challenging to perform a detailed study. Apart from location, we did not analyze other determinants of ESD duration and speed. However, we were able to clarify submucosal fibrosis and confirmed, for the first time upon dissection in the lesser curvature of the body, locations at which hemostasis can be difficult (e.g., the greater curvature of the body).

It has been reported that the prolongation of ESD duration increases the rate of complications [20, 21], and, thus, it is also very important to choose the lesion treatment with a clear expectation of the time requirements for each stage (e.g., incision and dissection) in consideration of the skill level of the operator.

## 5. Conclusion

In the present study, we compared CIS and SDS in ESD of gastric lesions and reported the CIS and SDS for different locations for the first time. On the basis of our results, we predicted the CIS and SDS according to different locations and clarified the underlying factors that affect procedural complexity and speed.

## Figures and Tables

**Table 1:** Baseline characteristics of gastric tumors.

Characteristics	Value	(%)
Sex (females : males)	80 : 222 (1 : 2.5)	
Age, years, median (range)	73 (40–92)	
Morphology		
Protruded (0-I)	11	(3.7)
Flat (0-II b, 0-II a)	143 (6, 137)	(47.4)
Depressed (0-II a + II c, 0-IIc)	147 (138, 9)	(48.8)
Submucosal tumor	1	(0.3)
Tumor size (mm), median (range)	11 (2–60)	
Specimen size (mm), median (range)	34 (14–110)	
Histology		
Adenoma	36	(11.9)
Differentiated type	247	(81.8)
Mix type (differentiated + undifferentiated type (<20 mm))	12	(4)
Undifferentiated type	5	(1.7)
Others^*∗*^	2	(0.7)
Ulcer or scar	24	(7.9)
Hidden fibrosis	35	(11.6)
Much time to hemostasis	51	(16.9)
Perforation	7	(2.3)
Delayed bleeding	9	(3.0)

^*∗*^Others: one case was neuroendocrine tumor and another was hyperplastic polyp.

**Table 2:** Classification of lesions by location (*n* = 302).

Location number	*n*	(%)
1: AEGJ	5	(1.7)
2: fornix	7	(2.3)
3: lesser curvature of the body	33	(10.9)
4: greater curvature of the body	21	(7.0)
5: anterior wall of the body	22	(7.3)
6: posterior wall of the body	46	(15.2)
7: across the angle	34	(11.3)
8: lesser curvature of the antrum	45	(14.9)
9: greater curvature of the antrum	17	(5.6)
10: anterior wall of the antrum	34	(11.3)
11: posterior wall of the antrum	34	(11.3)
12: APR	4	(1.3)

AEGJ: across the esophagogastric junction; APR: across the pyloric ring.

**Table 3:** Characteristics of the cases in the 12 locations.

Location	*n*	Mean age	Sex Females : males	Ulcer or scar (%)	Hidden fibrosis (%)	Much time to hemostasis (%)	Tumor size (mm), median	Circumference (mm), median	Resected area (mm^2^), median
1	5	64.4	1 : 4	1/5 (20)	1/5 (20)	3/5 (60)	12	206.9	3187.1
2	7	72.1	2 : 5	1/7 (14.3)	2/7 (28.6)	3/7 (42.9)	10.5	182.3	2637.6
3	33	72.4	7 : 26	2/33 (6.1)	8/33 (24.2)	10/33 (30.3)	13.5	195.3	2901.4
4	21	69.5	3 : 18	2/21 (9.5)	2/21 (9.5)	3/21 (14.3)	16	201.8	3165.1
5	22	72.4	4 : 18	1/22 (4.5)	2/22 (9.1)	5/22 (22.7)	14	200	3132.2
6	46	70.1	12 : 34	4/46 (8.7)	8/46 (17.4)	13/46 (28.3)	14.5	182.2	2637.6
7	34	74.1	10 : 24	4/34 (11.8)	6/34 (17.6)	9/34 (26.5)	18	226.8	4042.8
8	45	72.6	14 : 31	4/45 (8.9)	4/45 (8.9)	2/45 (4.4)	18.5	219.2	3733.5
9	17	71.5	3 : 14	2/17 (11.8)	1/17 (5.9)	0/17 (0)	10.5	182.5	2625
10	34	72.2	12 : 22	1/34 (2.9)	0/34 (0)	1/34 (2.9)	12	183.6	2512
11	34	74.4	9 : 25	1/34 (2.9)	0/34 (0)	2/34 (5.9)	12	173.1	2373.8
12	4	76	3 : 1	1/4 (25)	1/4 (25)	0/4 (0)	14	211.1	3504.2

*p* value		0.24					<0.05	<0.01	<0.01

1: the lesion across the esophagogastric junction (AEGJ); 2: fornix; 3: lesser curvature of the body; 4: greater curvature of the body; 5: anterior wall of the body; 6: posterior wall of the body; 7: the lesion across the angle; 8: lesser curvature of the antrum; 9: greater curvature of the antrum; 10: anterior wall of the antrum; 11: posterior wall of the antrum; 12: the lesion across the pylorus ring (APR).

**Table 4:** Mucosal circumference incision speed and submucosal dissection speed cut (speed per minute) in descending order.

Rank	Mucosal circumference incision speed			Submucosal dissection speed		
	Location	Median (mm/min) range	Interquartile range	Location	Median (mm^2^/min)	Interquartile range
1	9	19.3 (3.8–39.3)	18.3	10	214.6 (47.1–942)	174.5
2	10	18.0 (5.9–81.7)	10.9	9	204.1 (55.8–653.1)	309.3
3	11	15.6 (3.8–95.8)	13.7	11	175.1 (34.1–576.9)	201.2
4	8	13.9 (3.3–35.2)	11.3	8	118.2 (19.3–502.4)	114.1
5	4	12.8 (4.2–24.0)	8.6	3	116.7 (23.1–440.4)	159.4
6	3	12.4 (2.9–34.1)	10.7	5	116.0 (15.2–338.2)	92.7
7	5	11.5 (2.6–35.5)	10.4	4	96.4 (55.9–316.5)	61.2
8	2	10.0 (3.1–30.4)	12.5	6	93.5 (7.6–506.6)	80.1
9	7	9.2 (2.5–40.3)	8.15	7	92.5 (20.9–474.4)	125.9
10	6	8.2 (1.7–26.5)	6.6	1	91.1 (12.8–157)	97.7
11	1	7.6 (2.9–11.2)	6.4	2	66.6 (7.6–141.3)	49.2
12	12	4.5 (3.3–6.1)	2.2	12	43.5 (12.2–236.4)	170.9

*p* value		<0.01			<0.01	

**Table 5:** Subgroup analyses by procedure time for mucosal incision and submucosal dissection on ESD.

	Mucosal incision				Submucosal dissection			
	Location	Rate, median (range)	Interquartile range	*p* value	Location	Rate, median (range)	Interquartile range	*p* value
Fast group	8, 9, 10, 11	15.6 (3.3–95.8)	12		8, 9, 10, 11	170.5 (19.3–942.0)	178.7	
Moderate group	2, 3, 4, 5	12.4 (2.6–35.5)	10.6	*p* < 0.01	3, 4, 5, 6	97.7 (7.6–506.6)	79.7	*p* < 0.001
Late group	1, 6, 7, 12	8.2 (12.2–474.4)	6.7	*p* < 0.01	1, 2, 7, 12	89.2 (12.2–474.4)	90.4	0.42

1: the lesion across the esophagogastric junction (AEGJ); 2: fornix; 3: lesser curvature of the body; 4: greater curvature of the body; 5: anterior wall of the body; 6: posterior wall of the body; 7: the lesion across the angle; 8: lesser curvature of the antrum; 9: greater curvature of the antrum; 10: anterior wall of the antrum; 11: posterior wall of the antrum; 12: the lesion across the pylorus ring (APR).
